# Thyroid Langerhans cell histiocytosis concurrent with papillary thyroid carcinoma: A case report and literature review

**DOI:** 10.3389/fmed.2022.1105152

**Published:** 2023-01-19

**Authors:** Bin Mi, Di Wu, Yue Fan, Benjamin Ka Seng Thong, Yudong Chen, Xue Wang, Chaofu Wang

**Affiliations:** ^1^Department of Pathology, Pingyi County People’s Hospital, Linyi, China; ^2^Department of Pathology, Xuzhou Central Hospital, Xuzhou, China; ^3^Department of Pathology, Ruijin Hospital, Shanghai Jiao Tong University School of Medicine, Shanghai, China; ^4^Shanghai Jiao Tong University School of Medicine, Shanghai, China; ^5^Department of Ultrasound, Ruijin Hospital, School of Medicine, Shanghai Jiao Tong University, Shanghai, China

**Keywords:** papillary thyroid carcinoma (PTC), lymph node metastasis, Hashimoto’s thyroiditis (HT), *BRAF* gene mutations, Langerhans cell histiocytosis (LCH)

## Abstract

Langerhans cell histiocytosis (LCH) is a clonal neoplasm of myeloid dendritic cells, rarely involving the thyroid gland. Papillary thyroid carcinoma (PTC) is the most common histological subtype of thyroid cancer. We report a rare case of a 34-year-old Chinese woman who has LCH with PTC and cervical lymph node metastasis of LCH, with a review of the literature. The patient has thyroid nodules and cervical lymph node enlargement detected by neck ultrasound during physical examination. Fine needle aspiration cytology (FNAC) showed PTC with Hashimoto’s thyroiditis and *BRAF V600E* mutation after thyroidectomy and lymph node dissection. Histopathological examination suggests that LCH was concurrent with classical PTC, accompanied by LCH cervical lymph node metastasis. No *BRAF, HRAS*, and *TERT* promoter mutations were detected in LCH metastatic lesions. The patient is in stable clinical condition currently.

## Introduction

Langerhans cell histiocytosis (LCH) is a rare systemic disease characterized by clonal proliferation of CD1a +, Langerin + myeloid dendritic cells (1). Noticeably, LCH cells share similarities with antigen-presenting Langerhans cells, but they are derived from myeloid dendritic precursor cells (2). LCH can involve single or multiple systems, including bone, skin, pituitary gland, lymph nodes, liver, spleen, and lungs, with bone and skin as the most common sites of involvement. Clinical presentation and multisystem involvement determine the prognosis (3). LCH is most prevalent in children but rare in adults (4). Papillary thyroid carcinoma (PTC) is the most common type of thyroid malignant tumor. The detection rate of thyroid cancer has increased with the development of diagnostic imaging technology and fine needle aspiration cytology (FNAC) (5). To date, only 22 cases of LCH of thyroid concurrent with PTC have been reported, and only 11 cases of them had lymph node metastasis involving LCH. We present a case of single-system thyroid LCH concurrent with PTC, accompanied by lymph node metastasis involving LCH, and review the relevant literature to explore its pathological features.

## Case presentation

**FIGURE 1:**
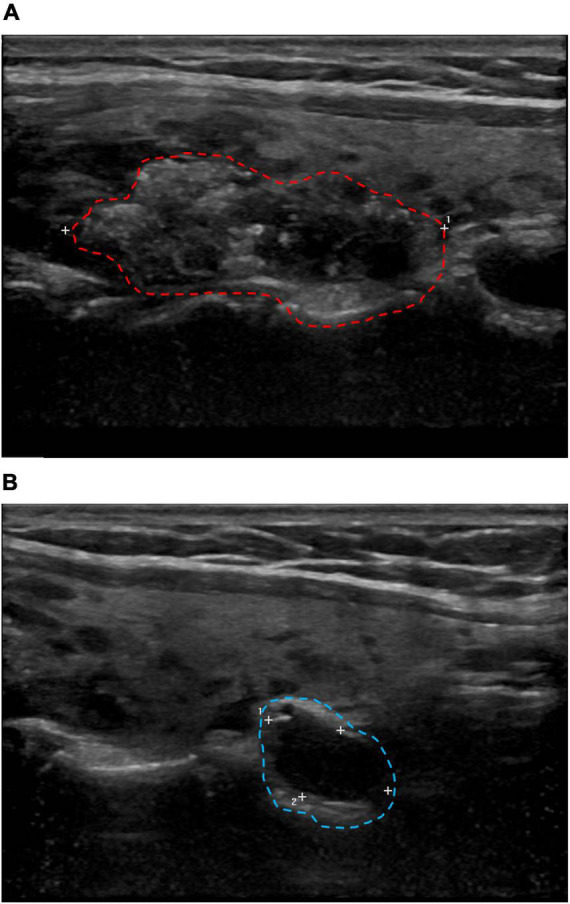
Cervical B-ultrasound. **(A)** Solid mass in the middle and upper pole of the right lobe of the thyroid gland, 33.4 × 14.5 × 21.2 mm (red circle). **(B)** The right cervical region VI lymph node showed enlargement, up to 12 × 6 mm (blue circle).

A 34-year-old female patient without a history of smoking or alcohol consumption showed no abnormalities on physical examination. Neck ultrasonography revealed a 33.4 mm × 14.5 mm × 21.2 mm solid mass in the middle-upper right lobe of the thyroid gland (Figure 1A) and an enlarged right cervical lymph node (12 × 6 mm) in level VI (Figure 1B). Blood thyroid function tests showed a significant increase in thyroglobulin, a decrease in calcitonin, normal thyroid stimulating hormone, free thyroxine, and free triiodothyronine levels. The FNAC suggested PTC with Hashimoto’s thyroiditis and *BRAF V600E* mutation.

**FIGURE 2:**
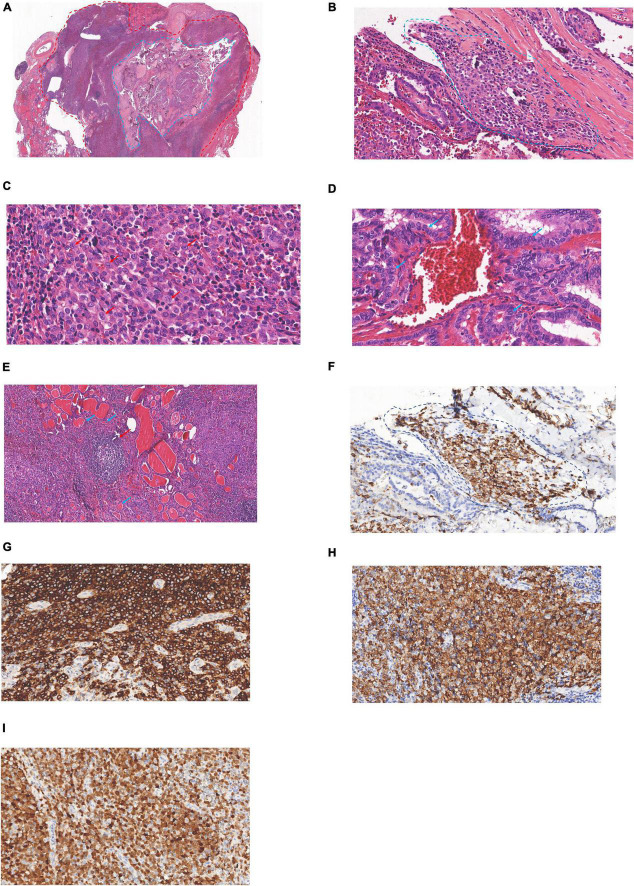
Langerhans cell histiocytosis (LCH) of the thyroid concurrent with PTC: **(A)** PTC region (blue circle), LCH region (red circle, 1 ×). **(B)** Junctional zone between PCT and LCH (blue circle, 20 ×). **(C)** LCH region (red arrow, 40 ×). **(D)** PCT region (blue arrow, 40 ×). **(E)** Hashimoto’s thyroiditis, lymphoid follicle (red arrow), atrophic thyroid follicles (blue arrow) (10 ×). **(F)** Langerin (+) indicated that LCH cells were found in the stroma of PTC (blue circle, 20 ×). **(G)** CD1a cytoplasmic/membrane + (20 ×). **(H)** Langerin cell membrane + (20 ×), **(I)** S-100 nucleus/cytoplasm + (20 ×).

The patient underwent total thyroidectomy and lymph node dissection of the right lobe. A gross examination of the thyroid revealed a hard and poorly circumscribed nodule (35 × 30 × 9 mm) in the middle-upper right lobe of the thyroid gland. In the low-power HE section, two regions of the thyroid lesions showed LCH and PTC (Figure 2A). The PTC section composed of slender, branched papillary structures, and scattered gravel bodies. The LCH area composed of epithelioid/histiocyte-like cells with abundant pink cytoplasm. The fusion of PTC and LCH was observed under medium power, and LCH cells were found in the papillary stroma of PTC (Figure 2B). LCH cells have abundant cytoplasm, are mildly eosinophilic, have epithelioid/histiocytoid appearance, have large and irregular nuclei, slightly atypical nuclei, and diverse morphology, with vacuolated or “coffee bean” appearance, inconspicuous nucleoli, and scattered eosinophilic infiltration in the stroma (Figure 2C). PTC cells were crowded, with ground-glass nuclei, large nuclei, irregular karyotypes, and visible nuclear furrows (Figure 2D). The remaining sections of the thyroid gland were Hashimoto’s thyroiditis (Figure 2E), with lymphoid follicle formation and atrophic changes in thyroid follicles. IHC examination determined that Langerin + LCH cells were present in the papillary stroma of PTC (Figure 2F) and CD1a +, langerin +, and S-100 + LCH cells were determined in the LCH region (Figures 2G–I). LCH areas showed a Ki-67 index of 40%. In PTC section, IHC determined AE1/AE3 +, TTF-1 +, CK19 +, HBME-1 +, Ki67 (10% +), and CD56- PTC cells.

**FIGURE 3:**
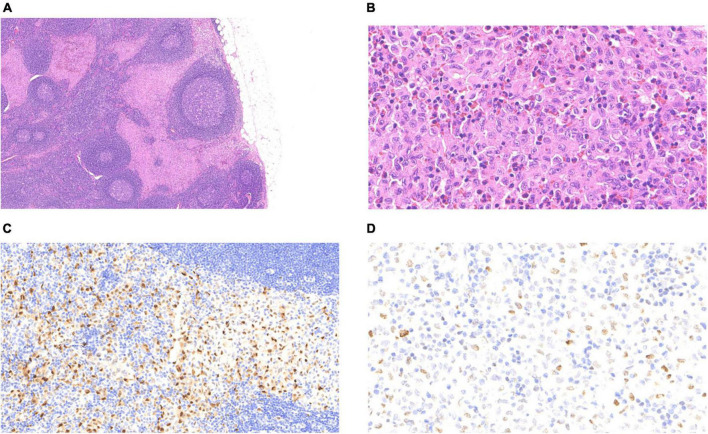
Langerhans cell histiocytosis of the lymph nodes: **(A)** The pink-stained area at the subcapsular sinus was that of LCH (4 ×). **(B)** LCH cells were diffusely distributed with eosinophils (40 ×). **(C)** Nuclear positivity for cyclin D1 (20 ×). **(D)** Variable nuclear positivity for p53 (40 ×).

The LCH metastasis was identified in the subcapsular sinus of the lymph node under low magnification (Figure 3A). At high magnification, LCH cells had more abundant eosinophilic cytoplasm, large and irregular nuclei, slightly atypical nuclei, and diverse morphology, with vacuolated or “coffee bean” -like appearance, and scattered eosinophils in the stroma (Figure 3B). No PTC metastasis was found in the lymph nodes. IHC staining confirmed CD1a/Langerin + and S-100 + LCH cells within the lymphatic sinuses. The differential expression of cyclin D1 and p53 helps to exclude reactive hyperplasia Langerhans cells, which was more supportive of LCH (Figures 3C, D). We performed PCR molecular testing of the LCH region in the lymph nodes, which did not detect *BRAF, TERT (C228T/C250T)* promoter, or *HRAS* mutations.

## Discussion

Primary thyroid LCH in adults is rare (4, 21, 23). PTC is the most common thyroid cancer, accounting for 80–90%, and is more common in women (14, 24). Table 1 summarizes 22 previous published cases and the present case. The age of patients ranged from 3 to 69 years (median = 37 years). Thyroid LCH with PTC was more prevalent in men (59.1% of the cases), and in adults, they were more common (9, 25, 26).

Thyroid LCH might be a multisystem disease. Multisystem examination and extended follow-up time are needed once diagnosed as LCH (9, 27). Of the 81.8% of LCH involved in multisystem, 50.0% involved the lymph nodes (Table 1). Therefore, reommend the necessity of multisystem examination and radiological examination such as chest CT scan, whole body bone scan, head MRI, and abdominal ultrasound for follow-up. The radiological findings of the first 3 months commonly present scattered multiple nodules with no significant changes in the bilateral lung. Noticeably, patients with LCH involving the lungs have a poor prognosis (27–29). Therefore, long-term follow-up is highly recommended for the current patient (14). LCH is closely associated with a variety of malignant tumors, including lung cancer and leukemia (6, 30). The current patient’s chest CT showed sporadic subpleural nodules in both lungs, which might suggest LCH involving the lungs. It is commonly seen in smokers, but pulmonary LCH can still be seen in non-smokers. The patient had no smoking habit, but chest CT was still strongly recommended in the future (31).

Fine needle aspiration cytology can preliminarily diagnose most thyroid diseases, but the diagnosis of LCH of the thyroid using FNAC is challenging due to the similarity in HE morphology between PTC cells and LCH cells (8, 32). In Table 1, only 64.7% of PTC, 41.2% of LCH, and 29.4% of LCH concurrent with PTC were identified among the 22 cases which underwent preoperative FNAC examination. Li et al. reported that the sensitivity of FNAC for the diagnosis of thyroid LCH was 37.5%, which was consistent with our study (22). Goldstein reported a case in which thyroid FNAC accurately distinguished LCH and PTC and proposed that PTC and LCH cells showed nuclear grooves, LCH cells had more granular cytoplasm, however, PTC showed a large number of cubic/columnar cells forming small papillary structures and produced a small amount of glia (6). The other diseases of LCH, such as lymphoma, Hashimoto’s thyroiditis, or undifferentiated carcinoma, are easily confused, which is challenging for pathologic examination. Morphologically, lymphoma cells usually have more basophilic cytoplasm. The lymphocyte component of Hashimoto’s thyroiditis was polyclonal. Undifferentiated carcinoma showed more cellular atypia. IHC such as CD1a and Langerin can be used to assist differential diagnosis (33).

**TABLE 1:** Summary of the literature review of concomitant Langerhans cell histiocytosis and papillary thyroid carcinoma.

References	Gender/Age	PTC and LCH in thyroid	Side	LCH in LN	Hashimoto’s thyroiditis	*BRAF* mutations	LCH in other organs	Treatment	Follow up (months)	FNAC	
										Thyroid	LN
Goldstein and Layfield (6)	F/31	Y	Left	NR	Y	NR	Bone, pituitary gland, lung, skin,	Surgery, prednisone, CT	DF (6)	PTC LCH	NE
Safali et al. (7)	M/51	Y	Right	Y	No	NR	No	Surgery	NR	NE	Metastatic carcinoma
Saiz and Bakotic (8)	M/43	Y	Left	NR	Y	NR	No	Surgery	DF (24)	PTC suspected	NE
Foulet-Rogé et al. (9)	F/42	Y	Left	NR	Y	NR	No	Surgery	DF (14)	NR	NR
Burnett et al. (10)	M/3	Y	LCH In right PTC In left	NR	Y	NR	Lung	Surgery, prednisone, CT	NR	LCH	NE
Jamaati et al. (11)	M/24	Y	Bilateral	NR	No (mixed inflammatory cell infiltration)	NR	Lung	Surgery, CT, dexamethasone	NR	PTC LCH	NE
Vergez et al. (12)	M/29	Y	Bilateral	NR	Y	NR	Bone, pituitary gland, lung, skin	Corticosteroids, CT	Died due to compression of trachea	LCH HT	NE
Dong Hae Chung et al. (13)	F/53	Y	Right	NR	Y	NR	NR	Surgery	NR	HT	NE
Ceyran et al. (14)	M/37	Y	Bilateral	Y	Y	NR	NR	Surgery	Died due to cardiac arrest	Variant of uncertain significance.	NE
Gordon and Gordon (15)	F/22	Y	Bilateral	NR	Y	*BRAF V600E* in PTC	Labia vulva	Surgery, Prednisone	NR	NR	NR
AlZahrani et al. (16)	F/27	Y	Bilateral	Y	Y	NR	No	Surgery, CT Prednisone	NR	PTC HT	NE
Wu et al. (17)	M/40	Y	Right	Y	NR	NR	Lung, Liver,	Surgery, CT	DF (24)	PTC	NE
Jaimanti Bakshi and Joshi Kiran (18)	M/31	Y	Right	NR	No (mixed inflammatory cell infiltration)	NR	No	Surgery, CT	DF (6)	LCH	NE
A Al Hamad et al. (19)	F/37	Y	Bilateral	Y	No	*BRAF V600E* in PTC, *BRAF V600K* in LCH	No	Surgery, prednisone CT	DF (24)	PTC	NE
Zaidi et al. (20)	M/31	Y	Right	NR	No	*BRAF V600E*	Multisystem LCH	Surgery, CT radioactive iodine treatment	DF (12)	PTC and LCH	NE
Ozisik et al. (5)	M/58	Y	Left	NR	No	NR	Pituitary gland	surgery	NR	NE	NE
	M/45	Y	LCH in right, PTC and LCH in left	Y	No	*BRAF V600E* in PTC and LCH	Pituitary gland gingiva	Surgery Radiotherapy (iodine)	Six months after surgery: LCH gingival infiltration	A malignant tumor is suspected.	PTC LCH
Maraqa et al. (21)	F/49	PTC only	PTC in right, LCH in left	Y	No	NR	Lung	Surgery	DF (3)	PTC	LCH
	M/69	PTC only	PTC and LCH in left	Y	No	NR	Bone marrow	Surgery	After 48 months, LCH involved the bone marrow	PTC	NE
Li et al. (22)	F/19	Y	NR	Y	NR	NR	Hypothalamic-pituitary axis breast	Surgery CT	DF (17)	NR	NR
	M/40	Y	NR	Y	NR	NR	Hypothalamic-pituitary axis lung, bone	Surgery CT	DF (33)	NR	NR
Current patient	F/34	Y	Right	Y	Y	No find *BRAF V600E, TERT, HRAS* in LCH *BRAF V600E* in PTC	No	Surgery	DF (3)	PTC	NE

F, female; M, male; Y, yes; CT, chemotherapy; NR, not recorded; DF, disease free; LN, lymph nodes; HT, Hashimoto’s thyroiditis; NE, not examined.

Histological diagnosis is more advantageous than a cytological diagnosis in pathological diagnosis. However, the histological diagnosis of LCH is difficult to distinguish with reactive hyperplasia of Langerhans cells due to the similarity in morphology and expression of CD1a and Langerin. It has been reported that CD31, cyclin D1, and p53 are helpful for the differential diagnosis of the two diseases (34–36). Cyclin D1 (+) and p53 (scattered +) supported the diagnosis of LCH in this case (Figures 3C, D).

Langerhans cell histiocytosis is closely associated with a wide variety of tumors such as lymphoma, leukemia, lung carcinoma, and other tumors (28, 30). However, the relationship and the mechanisms between LCH and PTC remain elusive due to the rarity of LCH of the thyroid concurrent with PTC. In HT, studies show that Th1 lymphocytes synthesize Interferon-gamma (IFN-γ) and tumor necrosis factor, which induce the thyroid gland cells to release CXCL10 and elicit autoimmunity. HT is closely related to PTC (37, 38). LCH is a tumor with inflammatory properties which provide a microenvironment for the formation of HT, subsequently increasing the risk of PTC (39–41). A percentage of 45.5 of the reported cases show the association of HT and LCH in terms of histological and FNAC examination. Therefore, we propose that LCH may be indirectly associated with PTC. Additionally, researchers also determined the hyperplasia of CD1a + dendritic cells in PTC, indicating that PTC provides a microenvironment for tumor transformation, leading to Langerhans cells undergoing clonal expansion and forming LCH (42). The aforementioned hypotheses support a pathogenic relationship between PTC and LCH.

*BRAF V600E* mutation is the most common molecular change among PTC and LCH. The mutation rate is 84.0% (4, 43, 44) and 25–64% for PTC and LCH, respectively (26). To date, only in 4 cases of PTC concurrent with LCH molecular analysis has been completed and only in 1 case *BRAF V600E* mutation has been determined. This suggests that *BRAF V600E* mutation might not contribute to the pathological process when PTC and LCH co-occur. In this case, *BRAF V600E* mutation was found in FNAC, but *BRAF, TERT*, and *HRAS* are not mutated in the LCH section in the lymph nodes. This suggests that *BRAF V600E* mutation occurs in PTC. The study also observed that *BRAF N486 P490* mutation rate is 57.1% in LCH patients with thyroid involvement. *BRAFN486 P490* mutations are found more commonly in LCH patients with thyroid involvement than those without thyroid involvement (45). This suggests that the molecular pathology in PTC concurrent with LCH warrants further research as it might provide clinical information. There is a great possibility of poor prognosis due to the co-occurrence of the two diseases. Thus, long-term follow-up is required for these patients.

## Conclusion

Thyroid LCH and PTC with LCH lymph node metastasis are rare. The application of FNAC in differentiating thyroid LCH and PTC is difficult. The diagnosis of LCH with PTC requires both a histological and an immunohistochemical examination. Thyroid LCH with PTC may indicate a poor prognosis, therefore, once diagnosed, it is recommended to improve relevant examinations and extend follow-up time.

## Data availability statement

The original contributions presented in this study are included in the article/supplementary material, further inquiries can be directed to the corresponding authors.

## Ethics statement

Written informed consent was obtained from the individual(s) for the publication of any potentially identifiable images or data included in this article.

## Author contributions

BM was the principal author of the manuscript. DW was responsible for picture collection and history collection. YF was responsible for immunohistochemical staining and interpretation of the results. BT was responsible for the language correction of the article. YC provided the ultrasound data. XW constructed the idea of the article and revised the article. CW was the last to review the article. All authors contributed to the article and approved the submitted version.
